# What Impedes General Practitioners’ Identification of Mental Disorders at Outpatient Departments? A Qualitative Study in Shanghai, China

**DOI:** 10.5334/aogh.2628

**Published:** 2019-11-11

**Authors:** Hanzhi Zhang, Dehua Yu, Zhaoxin Wang, Jianwei Shi, Jie Qian

**Affiliations:** 1 Department of General Practice, Yangpu Hospital, Tongji University School of Medicine, Shanghai, CN; 2 School of Public Health, Shanghai Jiaotong University School of Medicine, Shanghai, CN; 3 Shanghai General Practice and Community Health Development Research Center, Shanghai, CN; 4 Department of Psychology, Yangpu Hospital, Tongji University School of Medicine, Shanghai, CN

## Abstract

**Background:**

Mental disorders endanger people’s health and lives. General practitioners (GPs) play a valuable role in identifying and treating mental disorders in outpatient clinical settings. However, there are obstacles for GPs’ identification in developing countries.

**Objective:**

This study’s aim was to identify the related obstacles and to propose optimized strategies.

**Methods:**

We conducted qualitative interviews with 26 GPs from seven randomly sampled community healthcare centers in Shanghai, China. The interview guide was based primarily on the items from mental status evaluation. After transcribing, coding, condensing, and categorizing talking content, we summarized the theme structure and results.

**Findings:**

GPs lacked the confidence and skills to conduct psychiatric evaluation and seldom conducted it. Patients’ behaviors also influenced whether evaluations were conducted. The GPs expressed that they were short of strategies and wished to be well trained and have sufficient practice. We found that two major reasons impeded GPs’ identification. First, the GPs had difficulty making a diagnosis: they lacked diagnostic ability and confidence, they had misunderstandings about diagnoses, and they had unclear qualifications for making psychiatric diagnoses. Second, the GPs lacked skills for evaluation and reevaluation: their evaluation had inadequacies of contents and subjects; they lacked mental state examination evaluation, communication, and severity assessment skills and knowledge.

**Conclusions:**

This study found that it is difficult for GPs in developing countries to become competent in the diagnosis and systematic evaluation of mental disorders without external help. Unclear qualification also limited GPs’ diagnoses of mental disorders. We proposed that optimized strategies to overcome these challenges lie in support of changes in policy, programs, and utilizing effective tools, such as the mhGAP, GMHAT/PC, BVC, Grille’s assessment tool, and telemedicine.

## Background

Mental and behavioral disorders endanger people’s health and lives. General practitioners (GPs) play a valuable role in identifying and treating mental disorders. According to Jang, patients with mental disorders received higher rates of medical diagnoses and a greater number of psychiatric medications under the medical services of GPs and psychiatrists compared to patients only receiving mental health services from psychiatrists [1]. In China, Li and colleagues reported that integrated assessment and intervention conducted by Beijing general practice groups for patients with major mental disorders improved the complete self-care and independent living ability of the patients [2]. However, some studies suggested that there were considerable deficiencies in the process of identifying and managing mental disorder cases in general practice, which were particularly noticeable in developing counties, manifested by low diagnostic rate, lack of attention to psychological problems, and lack of confidence in diagnosis and treatment [3,4,5].

International articles, manuals and literature have proposed a variety of strategies to help GPs to identify patients with mental disorders [6,7,8,9]. For example, Blashki lists the items of mental state evaluation in *General Practice Psychiatry* [6]; manuals of the National Institute for Health and Clinical Excellence (NICE) and the U.S. Preventive Services Task Force (USPSTF) mention methods and suggestions for evaluating risks of suicide and violence [8,9]. Meanwhile, some studies focus on the cooperation between the general practice and psychiatry departments in order to enhance treatment [10,11,12,13]. Nevertheless, studies also reveal that identifying mental disorders at general practice outpatient departments is restricted by many factors, such as the GPs’ perceptions and actions, and the local medical resources and policies [5,14]. Moreover, current strategies are mentioned in guidelines and research of developed countries, where general practice is more advanced than in developing countries. Hence, the implication for developing countries is that in order to have suitable and optimized strategies, not only do they need to draw experience from developed countries, but they also need to analyze and integrate these with existing strategies. During this process, essential steps require us to understand the GPs’ perceptions and actions in identifying mental disorders and to find out what impedes their identification of mental disorders in outpatient departments. This is a fundamental step to help GPs improve their efficiency in identifying mental disorder cases and provide evidence for policy making. More importantly, the optimized strategies based on obstacle analysis will help developing countries whose medical resources are now limited and primary healthcare systems are still in primary stages.

As a developing county, general practice services in China are still in an early stage of development. We chose to interview GPs in Shanghai, where general practice is quickly developing and the GPs’ identification of mental disorders has more potential for improvement. Since 2014, Shanghai has taken the lead in launching the training of GPs’ mental and psychological skills in China. Although there are still many problems in training which can be improved upon, the teaching staff has been established [15]. In this study, face-to-face interviews were conducted with doctors in both urban and suburban community healthcare centers by random sampling. The purpose of this study is to understand the GPs’ perceptions and actions in identifying mental disorders; to identify the related obstacles; and to propose optimized strategies.

## Methods

### Research team

We had five members in our research team, two male and three female. Two members had master’s degrees, and the other three had doctorates. The team members included GPs, professors of public health, and a psychologist. The members as well as their departments had clinical and research collaborations with the community hospitals in Shanghai and GPs at those hospitals. The research team as a whole had previous experience in conducting qualitative interviews on public health in community healthcare centers.

In order to ensure the quality of the interviews, all staff who were involved met to confirm interview domains, questions, and the method of questioning.

### Respondent selection

A multi-region sample was obtained. To obtain a representative sample, we targeted three urban districts (Yangpu, Huangpu, Jingan) and one suburban district (Qingpu) by random sampling. We randomly selected two community healthcare centers from each downtown district and one hospital from the suburban district, with seven hospitals in total.

We conducted informational surveys with the outpatient GPs in the seven community healthcare centers before the interview. The survey included the number of outpatient GPs in each community, their title and their willingness to complete the interview.

Then we developed the following inclusion criteria and exclusion criteria. Inclusion criteria: GPs with professional titles at mid-level or above; awareness or serious attitude in identifying mental disorders; interest in this study; and agreement with the significance of this study. Exclusion criteria: qualifications or experiences as a psychiatrist or psychologist. GPs were screened based on these inclusion and exclusion criteria to identify GPs qualified to participate. Then we randomly selected four GPs as respondents from each downtown hospital and two from the suburban hospital, with 26 GPs in total. Finally, we convened the 26 GPs to a meeting and gave them a detailed introduction to the significance, purpose, methods and content of this study.

### Study design and data collection

A survey conducted with GPs in a community in Shanghai showed that the GPs lacked knowledge on mental and psychological aspects of mental disorders [4]. In order to better understand and discover the perceptions, actions and problems of GPs in identifying mental disorders in community outpatient clinics, we adopted the following design for the interview guide and for the questions used in the in-depth interview.

The interview guide covered two domains:

1. GPs’ obstacles and suggestions in identifying mental disorders at general-practice outpatient departments.
2. GPs’ perceptions and actions towards each assessment item as mentioned in Blashki’s General Practice Psychiatry (see Table 1) [6].

**Table 1:** Framework of the interview domains and questions.

Number	Domains	Questions

1	GPs’ overall obstacles in and suggestions for identifying mental disorders at general-practice outpatient departments	What difficulties do you have in identifying mental disorders in outpatient service?
		What are your suggestions for identifying mental disorders in outpatient service?
2	GPs’ perceptions (including obstacles and suggestions) and actions toward each assessment item at general-practice outpatient departments	
2.1	Screening of first-time patients	What are your opinions of this aspect of identification? What are the obstacles and your suggestions?
		How do you administer this item?
2.2	Collecting the psychiatric history of first-time patients	What are your opinions of this aspect of identification? What are the obstacles and your suggestions?
		How do you administer this item?
2.3	Psychiatric examinations with first-time patients	What are your opinions of this aspect of identification? What are the obstacles and your suggestions?
		How do you administer this item?
2.4	Physical examinations and diagnostic tests of first-time patients	What are your opinions of this aspect of identification? What are the obstacles and your suggestions?
		How do you administer this item?
2.5	Primary diagnosis and records of psychiatric history	What are your opinions of this aspect of identification? What are the obstacles and your suggestions?
		How do you administer this item?
2.6	Severity of first-time patients’ mental disorders	What are your opinions of this aspect of identification? What are the obstacles and your suggestions?
		How do you administer this item?
2.7	Evaluation of relapsed or chronic patients	What are your opinions of this aspect of identification? What are the obstacles and your suggestions?
		How do you administer this item?

This interview guide acted as the framework for asking questions in the interviews. The interview questions were open-ended. We encouraged the GPs interviewed to say anything about their perceptions (including obstacles and suggestions) and actions in identifying mental disorders in community outpatient clinics. The specific interview framework and issues are illustrated in Table 1.

In-depth face-to-face qualitative interviews were conducted from November to December in 2018 by our research team. Our research team members interviewed GPs on a one-to-one basis in each community healthcare center.

To get detailed and reliable data, each interview lasted about one hour and was audio recorded. The audio records were returned to the respondents for confirmation after each interview.

The interview question data came to be saturated after we interviewed 26 respondents and obtained detailed information on similar problems in identifying mental disorders. The 26 respondents were coded with letters from A to Z. We recorded their genders, ages, titles, education, hospital areas, and related training information on whether they received prior psychiatric or psychological training.

### Data analysis

After all of the interviews were completed, we converted the recorded audio materials into electronic documents. After reading all of the files carefully, four members (HZZ, ZXW, JWS and JQ) of our research team coded the data. First, we conducted first-level coding of the answer to each question in the interview to familiarize ourselves with its corresponding content. Second, we repeatedly discussed and distilled the content, then developed second-level descriptive labels as codes and classified them. Finally, we summarized four major themes and their related minor themes. We selected representative quotations from the interview transcripts to highlight these themes, which are displayed in the results.

## Results

### Background information of the respondents

The number of GPs in the selected community healthcare centers, their professional titles and their intention to participate in research are shown in Table 2. The gender, working years, professional titles, academic qualifications, and community areas of the interviewed GPs are shown in Table 3.

**Table 2:** Information of GPs in the selected communities.

Hospital Code	Number of GPs	Professional titles of GPs			Intention to participate in research	

		Senior level	Mid-level	Junior level	Willing	Unwilling

H1	10	1	8	1	10	0
H2	15	3	11	1	15	0
H3	20	3	17	0	20	0
H4	10	2	8	0	10	0
H5	12	3	9	0	12	0
H6	15	4	11	0	15	0
H7	7	1	5	1	6	1
Total	89	17	69	3	88	1

**Table 3:** Characteristics of respondents who participated in the qualitative study (n = 26).

Characteristics	Number of respondents

Gender	
Male	10
Female	16
Working years	
≥5, <10	7
≥10, <20	13
≥20, <30	1
≥30, <40	5
Professional title	
Chief physician (Mid-level)	19
Associate chief physician (Senior level)	7
Academic qualification	
Undergraduate	8
Graduate	18
Community	
Urban	24
Suburban	2

Among the 26 GPs selected for the interview, one participated in the training of national psychological counselors and obtained the certificate. The remaining 25 GPs only participated in the psychology knowledge and training lectures organized by their own institutions and regional health authority they were affiliated with. The GPs applied the training through their hospitals with the goal to improve the knowledge and skills on mental health and psychology.

## Themes and results

We developed major and minor themes based on the interview data. Representative quotations from the interview transcripts for each major and related minor theme are as follows:

**Theme 1.** The GPs lacked confidence and skills of psychiatric evaluation and they seldom conducted it. Patients’ behaviors also influenced whether evaluations were conducted.

“I never learned about psychological knowledge and skills. I am not confident enough to deal with such kinds of patients. I don’t conduct psychiatric evaluation very often. Most of the patients with mental disorders come to the clinic repeatedly for prescriptions. They are usually in a rush. With other patients waiting and urging, it is hard to conduct mental evaluation.” (Respondent N)

**Theme 2.** The GPs expressed that they were short of strategies for identifying and treating mental disorders. They wished to be well trained and get sufficient practice.

“I need to receive more strategies and update my knowledge to identify and handle common mental disorders. I hope there would be training to enhance my knowledge about psychiatry. What’s more important is to gain experience by applying the knowledge into practice.” (Respondent Z)

**Theme 3.** The GPs had difficulties in making a diagnosis within three related minor themes.

1. The GPs lacked diagnostic ability and confidence.
  “Even though I believe that I am capable of identifying new cases of mental disorder patients, I find it hard to make diagnosis by myself. Besides, when some old patients fail to provide their former medical records and come here merely for prescription. I would have doubts about the diagnosis and prescriptions. But I don’t dare to make judgments independently.” (Respondent L)
2. The GPs had misunderstandings about diagnoses and screening procedures and were unaware of the risks.
  “I don’t screen patients with mental disorders in the outpatient department. But I think it is useful and with no risks. I hope there would be ready-made, handy scales of screening for early diagnosis. It will help anxious patients out of repetitive examinations, relieve their symptoms and provide them better early treatments.” (Respondent W)
3. The GPs had unclear qualifications for making psychiatric diagnoses.
  “Because I am not certainly qualified to make psychiatric diagnosis, I will follow the previous diagnosis. Most patients are not first-visit in community.” (Respondent B)
  “Though I learned about relevant knowledge, I believe mental issues may be beyond my duty and ability. Normally it depends, if it is not a busy day, I would probably chat a bit longer with the patient to see his/her mental state. But the clinic privacy is not good enough.” (Respondent F)

**Theme 4.** The GPs had absent functions of evaluation of four minor subthemes.

1. The GPs lacked systematic evaluation for patients of mental disorders. Their evaluation had inadequate content and subjects.
  “I ask about family condition, but not so much about other social backgrounds. If I do know information about social background, I hear of it occasionally from the patients’ colleagues or other people. Once I asked a patient with mental issues about her family, but not in detail. It involves the frictions between her as a daughter-in-law from other regions and her mother-in-law as a local resident.” (Respondent K)
  “I don’t evaluate mental state of children and perinatal pregnant women. Normally they go to community women and children healthcare clinic or general hospitals. But as far as I know, the community women and children healthcare clinic does not do much mental evaluation either.” (Respondent C)
2. The GPs lack systematic reevaluation for patients of mental disorders.
  “There is no systematic reevaluation. Most of the patients come here for prescriptions. If a doctor senses a significant change of illness, he/she will suggest the patient to go to the psychiatric department for treatments.” (Respondent D)
3. Although GPs had no obstacles performing body examinations, they lacked evaluating skills of mental state examinations and communication.
  “There is no difficulty in body examinations and laboratory testings. But as for the details of psychiatric examination content, I don’t know much about them. I just know there are facial expressions, movements, behaviors, etc. Common mental diseases such as anxiety and depression patients don’t normally show emotional symptoms in the outpatient department.” (Respondent A)
  “I don’t know how to communicate with patients with cognitive problems, let alone evaluate their mental state. I never received any training on this. Also, I think the patients consider psychiatric symptoms or disorders as taboo.” (Respondent H)
4. The GP lacked evaluating skills of severity assessment. They had no regular use of scales. They had misconceptions about violence and concerns about suicide.
  “I seldom evaluate the severity of disease and risk of violence. I rarely spot patients with suicidal tendencies. I think public health specialists are responsible for follow-up visits of this kind of patients. I have no idea of related evaluation and preventive measures. I had come across patients with anxiety or depression. I make judgments based on my experience. There is no handy scale for use and not enough time for evaluation.” (Respondent Q)
  “Although I learned about psychological knowledge, and I have a second-level psychologist certification, I hardly use scales in the general practice outpatient department. Usually I suggest patients to go to the psychiatric department for treatment.” (Respondent F)
  “There was once a patient showed violent actions because he/she was not satisfied with the prescription. There was no follow-up evaluation after the incident. The medical service section handles this kind of events.” (Respondent R)
  “I worry about the patients who take sleeping pills chronically. Meanwhile I can find nowhere to start with patients with suicidal history. I am worried that my intervention will exacerbate the suicidal risk.” (Respondent T)

## Discussion

From this study, we find that GPs had difficulties in both diagnosis and evaluation of patients with mental disorders at general outpatient departments. Below, we further analyze the reasons, and propose relative strategies for developing countries to resolve these problems.

It was found that unclear qualification limited GPs’ diagnosis of mental disorders. We posit that strategies to resolve this limitation lie in policy and program supports.

In this study, the respondents said they were not qualified to make psychiatric diagnoses and some of them felt it was not their duty to evaluate a patient’s mental state. Verhaak’s research revealed that GPs’ functions are related to multiple factors [14]. In gatekeeping countries such as the Netherlands, Spain and the United Kingdom, GPs made more psychological diagnoses. However, evaluation was more positive in non-gatekeeping countries [14]. This suggests GPs have ambiguous and missing qualifications in diagnosing mental disorders in developing countries, where primary healthcare systems are still in primary stages and GPs’ roles as gatekeepers are still unclear.

Moreover, in this interview, Respondent N said, “Most of the patients with mental disorders came to the clinic repeatedly for prescriptions. The patients were usually in a rush.” Also, Respondent F said, “The clinic privacy is not good enough for diagnosing patients with mental disorders.” This suggests that GPs’ unclear qualification also influences the outpatient environment and patient compliance, which limits GPs’ practice of diagnosis and increases its difficulty.

Changes in policy and program supports are needed to make GPs’ qualification of diagnosis clear. For example, Dutch GPs are no longer allowed to refer patients without a psychiatric disorder for mental health care since a 2014 reform of Dutch mental health care. More specifically, patients with non-complex psychological problems should be recognized and treated within general practice [16]. This observational case study found that the GPs reorganized mental healthcare in line with upcoming policy [16]. However, given that GPs lack the knowledge, ability and confidence in identifying mental disorders in developing countries [4,5], the changes in policy and program support should focus both on improving the training and practice of mental disorder diagnostic procedures.

The Mental Health Gap Action Program (mhGAP) offered by the World Health Organization (WHO) provides process paths based on symptoms for the diagnosis and treatment of common mental disorders for non-psychiatrists [7]. Siriwardhana suggested that a learning program of mhGAP improved general practitioners’ cognition about psychiatry [17]. Gureje and his colleagues trained general practitioners with an mhGAP project, which intended to strengthen their knowledge and skills concerning depression, neurosis, epilepsy, and alcohol abuse. Their results indicated that this training led to better performance on treating and referring mental disorder and substance abuse cases [7].

Mental disorders are usually concealed, repeated, and related to physical and social factors. Therefore, it is difficult for GPs in developing countries to become competent in the diagnosis and evaluation of mental disorders without external help. Strategies to circumvent this challenge lie in the use of effective tools and telemedicine.

Mental disorders are difficult to diagnose, especially in the early stages of disorders. Stigma also keeps patients from coming to the clinic for help. In this interview, Respondent A felt that patients with common mental disorders, such as anxiety and depression, did not typically illustrate emotional symptoms in the outpatient clinical setting. Respondent H thought that patients considered psychiatric symptoms or disorders as taboo. Jaruseviciene’s study shows that only 16.8% of the Lithuanian GPs questioned believe they have sufficient communication skills [5]. Another study by Harding showed that when faced with patients who speak a different language or come from a different cultural background, Australian GPs feel it is hard to communicate and make a diagnosis [18]. These studies indicate further difficulties for GPs’ identification, and that more mental disorder identification skills are needed. However, from this study, we also find that respondents lacked the skills required to perform mental state examinations. This indicates that in addition to more clinical skills training, GPs need more effective tools to help complete their diagnoses.

In the interview, Respondent W communicated that he hoped there would be ready-made, handy screening scales for early diagnosis. However, there has been no evidence to suggest that screening can help GPs perform efficient diagnoses even when using the common scale called the General Health Questionnaire (GHQ) [6]. Furukawa’s data of screening scales contrasts with Patel’s [19,20], which implied that screening scales increase the risk of overdiagnosing patients. This suggests that GPs should correct their misunderstandings about these diagnosis tools.

From this study, we also find that the respondents had no regular use of assessments to evaluate the severity of mental disorders. They also had misconceptions about violence and concerns about suicide. This indicate that GPs should learn how to choose suitable assessment tools. Tejada et al. found that the primary care and general health setting versions of the global mental health assessment tool (GMHAT/PC) accurately detected mental disorders, with a high level of sensitivity (81%) and specificity (92%) [21]. Grille’s assessment tool has been reported to perform better than qualitative interviews and assessments that are normally applied with a lower level of specificity in suicidal behaviors or risks [22]. Broset Violence Checklist (BVC) functions better than single non-structured clinical judgments, which was mentioned in the *NICE guidelines* [8].

Mental disorders are repeated and related to physical and social factors. Buszewicz et al. suggested that an all-around evaluation and intervention for patients with chronic depression in primary care would lead to a cost-effective improvement in medical and social outcomes when compared with care typically provided by GPs [23]. However, the results from the present study indicated that GPs’ evaluations lacked depth and comprehensiveness in biological, psychological, and social aspects. They also neglected evaluation for different kinds of visitors in the community, such as children, chronic patients, suicidal patients, and so on. This indicates that despite diagnosis, GPs should also improve their systematic evaluation methods.

Blashki lists relatively comprehensive items for psychiatric evaluation and reevaluation in *General Practice Psychiatry* [6]. These items are generally consistent with items of adult psychiatric evaluation from the *American Psychiatric Association Practice (APA) Guidelines (2015)* and those of adolescent and children psychiatric evaluation listed by E. Schaffalitzky [24,25]. *APA Guidelines (2015)* also points out that the evaluation could take more than one meeting to be completed, depending on the complexity of the symptom, the clinical setting, and the patient’s compliance, the ability to cooperate, and so on [24].

In the present study, GPs also said that patients who made repeated visits to community health centers did not visit a consistent doctor. This suggests that more strategies are needed in support of GPs’ systematic evaluation across services provided by different doctors. The NICE guidelines recommended sharing records for patients with mental disorders in primary care [8]. Fortney et al. investigated the telepsychiatry integration of mental health services into rural primary care settings [26]. This study mentioned two fundamental challenges in the mental healthcare system in the USA: a lack of capacity and an inequitable geographic distribution of services [26]. Because the primary care health system in developing countries faces similar problems [4,5], this suggests that strengthening the construction of telemedicine may be a beneficial strategy for developing countries to improve mental health recording and evaluation in primary care.

There are limitations in our study. First, in this qualitative study, we interviewed 26 GPs in Shanghai. It is necessary for us to do further quantitative surveys with a designed questionnaire in a large GP sample based on the detailed problems we found in the interview study. Second, given that most strategies, such as programs and assessments, in this study are proposed by the developed countries, it is important to note that further research is needed to determine the application of these strategies and adjustments required in different developing countries. Third, in this study, the respondents reflected that they had limited psychiatric supports at general practice outpatient departments. However, it is reported that cooperation between the departments of general practice and psychiatry holds great value in promoting treatment for patients of mental disorders, developing new skills for primary-care professionals and optimizing the arrangement of medical resources between these departments [10,11,12,13,27]. Further study regarding GPs’ perceptions and actions of this inter-departmental cooperation is needed. This may help to find more optimized strategies to improve GPs’ mental disorder diagnostic and evaluation skills.

## Conclusion

Mental disorders are usually concealed, repeated and related to physical and social factors. Thus, it is difficult for GPs in developing countries to become competent in the diagnosis and systematic evaluation of mental disorders without external help. Unclear qualifications also limited GPs’ diagnoses of mental disorders. We proposed that optimal strategies to solve these clinical challenges lie in support of changes in policy, programs, and utilizing effective tools, such as the mhGAP, GMHAT/PC, BVC, Grille’s assessment tool, and telemedicine.

## Data Accessibility Statement

The datasets generated and/or analyzed during the current study are not publicly available, as analysis of them is ongoing, but they are available from the corresponding author upon reasonable request.

## Additional File

The additional file for this article can be found as follows:

10.5334/aogh.2628.s1 Table. Codes and theme structure of the interviews.

## Abbreviations

General practitioner: GP

Mental Health Gap Action Program: mhGAP

National Institute for Health and Clinical Excellence: NICE

U.S. Preventive Services Task Force: USPSTF

General Health Questionnaire: GHQ

Broset Violence Checklist: BVC
