# Supplementary material for: What Impedes General Practitioners’ Identification of Mental Disorders at Outpatient Departments? A Qualitative Study in Shanghai, China
Source: Ann Glob Health. 2019 Nov 11;85(1):134. doi: 10.5334/aogh.2628 (PMC6857524; doi:10.5334/aogh.2628)
Supplement: Table. — Codes and theme structure of the interviews. [file agh-85-1-2628-s1.pdf]

Table

Codes and theme structure of the interviews

Number	Domains	Questions	Codes of the answer to each question	Descriptive labels (Code 2)	Theme and quotation
1	GPs' overall obstacles in and suggestions for identifying mental disorders at general-practice outpatient departments	What difficulties do you have in identifying mental disorders in outpatient service?	○	lack actions	1. The GPs lacked confidence and skills of psychiatric evaluation. They seldom conducted it. Patients' behaviors also influenced whether evaluations were conducted. *(Respondent N)*
				patients' behaviors	
				rely on experience	
				lack confidence	
				lack knowledge	
				lack strategies	2. The GPs expressed that they were short of strategies. They wished to be well trained and get sufficient practice. (Respondent Z)<br><br>3.The GPs had difficulties in

		What are your suggestions for identifying mental disorders in outpatient service?	◉	need training	making diagnosis.<br>(1)The GPs lacked diagnostic ability and confidence. (Respondent L)
				need knowledge	
				need practice	(2) The GPs had misunderstanding about diagnoses and screening procedures and were unaware of the risks. *(Respondent W)*<br>(3)The GPs had unclear qualification for making psychiatric diagnoses. (Respondents B, F)
2	GPs' perceptions (including obstacles and suggestions) and actions towards each assessment item at general-practice outpatient departments				
2.1	Screening of first-time patients	What are your opinions of this aspect of identification? What are the obstacles and your suggestions?	◆①	help to diagnose efficiently	
				no risk	4.The GPs had absent functions of evaluation.
		How do you administer this item?	★①	don't use scales to screen	(1) The GPs lacked systematic evaluation for patients of mental disorders. Their evaluation had inadequacies of contents and subjects. (Respondents K, C)
				don't screen	
2.2	Collecting the psychiatric history of first-time patients	What are your opinions of this aspect of identification? What are the obstacles	◆②	difficulties in communicating with patients and evaluating	(2)The GPs lack systematic reevaluation for patients of mental disorders. (Respondent D)<br>(3) Although GPs had no obstacle

		and your suggestions?			performing body examinations, they lacked evaluating skills of mental state examinations and communications. (Respondents A, H)<br>(4)The GP lacked evaluating skills of severity assessment. They had no regular use of scales. They had misconception about violence and concerns about suicide. (Respondents Q, F, R, T)
		How do you administer this item?	★②	limited and superficial issues	
				covered a limited variety of population	
2.3	Psychiatric examinations with first-time patients	What are your opinions of this aspect of identification? What are the obstacles and your suggestions?	◆③	know little	
		How do you administer this item?	★③	seldom do psychiatric examinations	
2.4	Physical examinations and diagnostic tests of first-time patients	What are your opinion of this aspect of identification? What are the obstacles and your suggestions?	◆④	Confident	
		How do you administer this item?	★④	no problem	
2.5	Primary diagnosis and records of psychiatric history	What are your opinions of this aspect of identification part? What are the obstacles and	◆⑤	unable to make psychiatric diagnosis independently	
				not certainly qualified	

		your suggestions?		Doubts	
				Difficulties	
		How do you administer this item?	★⑤	lack actions	
				absent records	
				incomplete records	
2.6	Severity of first-time patients' mental disorders	What are your opinions of this aspect of identification ? What are the obstacles and your suggestions?	◆⑥	know little	
				confuse medical disputes with violent behaviors	
				worry about patients' suicidal risk as well as the consequence of evaluation	
				no handy scale for use	
				time limitation	
		How do you administer this item?	★⑥	Lack action	
				seldom use scales	

2.7	Evaluation of relapsed or chronic patients	What are your opinions of this aspect of identification? What are the obstacles and your suggestions?	◆⑦	know little	
		How do you administer this item?	★⑦	lack systematic reevaluation	
				patients' behaviors	
				patients' compliance	
